# Ultrasound-Guided Hydrodissection for Sural Neuropathy After Calcaneus Fracture Surgery: A Case Report

**DOI:** 10.7759/cureus.47749

**Published:** 2023-10-26

**Authors:** Toru Omodani, Kenji Takahashi

**Affiliations:** 1 Orthopaedics, Tokyo Advanced Orthopaedics, Tokyo, JPN; 2 Sports Medicine and Joint Center, Funabashi Orthopaedic Hospital, Funabashi, JPN

**Keywords:** surgery, calcaneus fracture, sural neuropathy, ultrasound, hydrodissection

## Abstract

We present a case study of a 61-year-old man who experienced sural neuropathy following calcaneus fracture surgery, which was effectively treated using ultrasound-guided hydrodissection. Postoperatively, while the patient exhibited good bony fusion, he reported pain on the lateral side of the calcaneus. Ultrasound findings did not suggest any nerve discontinuity, but localized tenderness around the sural nerve was observed. After hydrodissection using 0.09% lidocaine, the patient's pain significantly decreased. Although hydrodissection alleviated the pain, complete resolution was achieved only post plate removal and neurolysis. This study represents the first report on the efficacy of hydrodissection for postoperative sural neuropathy, suggesting its potential as an effective treatment option.

## Introduction

The sural nerve is derived from either the medial or lateral sural cutaneous nerve, or both, and it runs distally down the posterolateral side of the ankle to the lateral foot [1]. Sural neuropathy is not a common condition, but it can occur in a variety of circumstances, including post-leg blood vessel surgery, peripheral nerve tumor, or trauma [2]. Yuebing et al. reported 36 cases of sural neuropathy, half of which were due to trauma or surgery [3]. Traumatic sural neuropathy can be caused by chronic causes, such as repeated ankle sprains or chronic external pressure from wearing tight shoes, or by a single episode, such as an ankle fracture or Achilles tendon repair [3,4].

In recent years, an increasing number of reports have suggested the efficacy of hydrodissection, a type of ultrasound-guided injection [5]. However, no reports have yet described the effect of hydrodissection on postoperative sural neuropathy of the ankle. We report a case in which hydrodissection was effective for sural neuropathy following calcaneal fracture surgery.

## Case presentation

**Figure 1:**
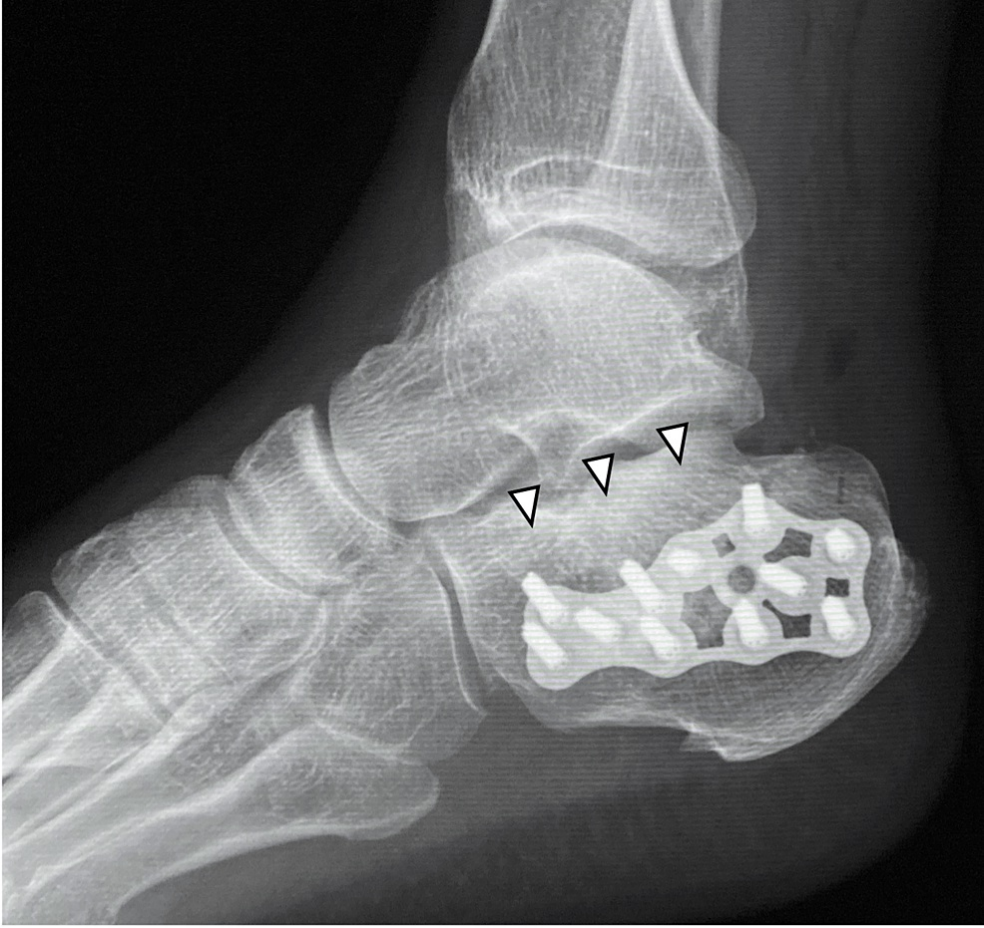
Radiograph. At five months postoperatively, good bony union (arrowheads) of the calcaneus fracture had been achieved.

A 61-year-old man underwent plate fixation using an extensile lateral approach for a calcaneal fracture. At five months postoperatively, good bony union was achieved, but she was limping due to pain in the lateral side of the calcaneus (Figure 1).

Ultrasound-guided palpation of the painful area revealed well-defined localized tenderness, consistent with that of the sural nerve. There was no ultrasound finding of nerve discontinuity suggestive of nerve tear.

**Figure 2:**
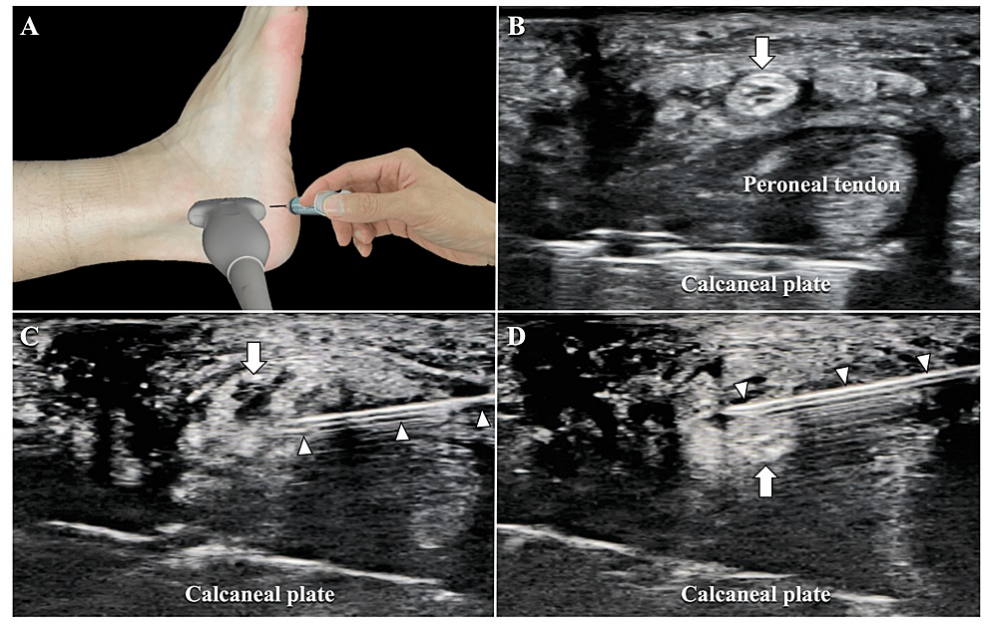
Ultrasound-guided sural nerve hydrodissection. An ultrasound probe was placed on the lateral side of the calcaneus, and the needle was inserted from the distal end (A). The ultrasound image depicts the peroneal nerve, which is located more superficially than the peroneal tendon (B). The tip of the needle was inserted into the deep (C) and superficial (D) layers of the sural nerve, and a total of 5 mL of 0.09% lidocaine diluted with saline was injected. Arrow: sural nerve; arrowheads: needle.

Ultrasound-guided hydrodissection of the sural nerve was performed at the site of the most tenderness. With the sural nerve scanned along the short axis, fluid dissection of the sural nerve was performed in both the deep and superficial layers under ultrasound guidance with a 25-gauge needle, using 5 mL of 0.09% lidocaine diluted in saline (Figure 2). The ultrasound imaging system used was a SONIMAGE HS1 with a linear probe L18-4 (Konica Minolta, Tokyo, Japan).

Immediately after hydrodissection, the pain improved from 10 to 3 on the numerical rating scale, and the patient's gait became normal. Plate removal and neurolysis were considered, but due to the COVID-19 pandemic, surgery could not be performed. Nevertheless, 10 months after the injection, the pain did not worsen. After the COVID-19 pandemic subsided, the plate was removed and neurolysis was performed. Firm scar tissue was developed around the nerve. The area between the scar tissue and the nerve was completely dissected. After surgery, the pain disappeared completely.

## Discussion

In this case, hydrodissection of the sural nerve was performed on the patient following plate fixation of a calcaneal fracture. The patient experienced an improvement in pain after hydrodissection.

Hydrodissection is a procedure in which a drug solution is injected mainly around peripheral nerves under ultrasound guidance [5]. The effectiveness of hydrodissection for peripheral nerves, such as the brachial plexus and median nerve, has been reported [6,7]. However, there have been no reports of sural nerve hydrodissection for residual pain after ankle surgery, and this study appears to be the first one.

Hydrodissection of the sural nerve was effective in a patient with lateral calcaneal pain after plate fixation of the calcaneal fracture. Internal fixation of calcaneal fractures with an extensile lateral approach is associated with sural neuropathy in up to 15% of cases [8]. When neuropathy occurs, conservative treatment includes shoe insoles or cups, physical therapy, and, if not successful, surgical procedures such as neurolysis or nerve decompression may be considered as treatment options [9].

A biomechanical study has demonstrated that performing hydrodissection on nerves improves their gliding resistance [10]. In addition to reducing the gliding resistance of nerves, hydrodissection is believed to be effective in alleviating pain originating from nerves by improving local circulation around the nerve [5]. In our case, pain improved after the hydrodissection of the sural nerve. However, although the pain improved after hydrodissection, it did not disappear completely. The pain completely resolved after plate removal and neurolysis. Thus, it appears that hydrodissection alone may be insufficient to resolve symptoms in the presence of firm adhesions.

In this case, 0.09% lidocaine was used as an injectable solution, and the effect of hydrodissection with pure saline could not be observed. This is due to the difficulty of performing hydrodissection with a solution that does not contain a local anesthetic, from the standpoint of the Japanese healthcare services provided by public health insurance. However, the fact that the injections were long-lasting and the symptoms did not recur even when a short-acting local anesthetic was used in a low-concentration diluted solution suggests that the effect of the hydrodissection itself may have been more effective than the effect of the local anesthetic. Despite these limitations, this report is novel in that it presents a new treatment option for sural neuropathy after calcaneus fracture surgery.

## Conclusions

In this report, hydrodissection of the sural nerve was performed on a patient following plate fixation of a calcaneal fracture. The patient experienced a significant improvement in pain. Hydrodissection for postoperative sural neuropathy has not been previously reported, making this study the first report of such a procedure. Our result suggests that hydrodissection may be an effective treatment option for postoperative sural neuropathy.
